# Effects of varying nursery phase-feeding programs on growth performance of pigs during the nursery and subsequent grow-finish phases

**DOI:** 10.1186/2055-0391-56-24

**Published:** 2014-11-06

**Authors:** Chai Hyun Lee, Dae-Yun Jung, Man Jong Park, C Young Lee

**Affiliations:** Department of Animal Resources Technology, Gyeongnam National University of Science and Technology, Jinju, 660-758 South Korea; The Regional Animal Industry Center, Gyeongnam National University of Science and Technology, Jinju, 660-758 South Korea

**Keywords:** Pig, Nursery, Feeding program, Growth, Grow-finish phase

## Abstract

The present study investigated the effects of varying durations of nursery diets differing in percentages of milk products on growth performance of pigs during the nursery phase (NP) and subsequent grow-finish phase (GFP) to find the feasibility of reducing the use of nursery diets containing costly milk products. A total of 204 21-d-old weanling female and castrated male pigs were subjected to one of three nursery phase feeding programs differing in durations on the NP 1 and 2 and GFP diets containing 20%, 7%, and 0% lacrosse and 35%, 8%, and 0% dried whey, respectively, in 6 pens (experimental units) for 33 d: HIGH (NP 1, 2 and 3 diets for 7, 14, and 12 d), MEDIUM (NP 2 and 3 for 14 and 19 d), and LOW (NP 2 and 3 and GFP 1 for 7, 14, and 12 d). Subsequently, 84 randomly selected pigs [14 pigs (replicates)/pen] were fed the GFP 1, 2 and 3 diets during d 54-96, 96-135, and 135-182 of age, respectively. The final body weight (BW) and average daily gain (ADG) of nursery pigs did not differ among the HIGH, MEDIUM, and LOW groups (14.8, 13.3, and 13.7 kg in BW and 273, 225, and 237 g in ADG, respectively). The average daily feed intake during the nursery phase was greater (p <0.01) in the HIGH group than in the MEDIUM and LOW groups, whereas the gain:feed ratio did not differ across the treatments. The BW on d 182 and ADG during d 54-182 were greater in the HIGH and MEDIUM groups vs. the LOW group (110.0, 107.6, and 99.6 kg in BW, respectively; p <0.01). The backfat thickness and carcass grade at slaughter on d 183 did not differ across the treatments. In conclusion, the MEDIUM program may be inferior to the commonly used HIGH program in supporting nursery pig growth. Nevertheless, the former appears to be more efficient than the latter in production cost per market pig whereas the LOW program is thought to be inefficient because of its negative effect on post-nursery pig growth.

## Background

Weaning is a most stressful transition not only socially but also nutritionally in the life of commercial pigs [1, 2]. Therefore, weaner pigs are usually provided with palatable diets containing highly digestible milk products such as lactose and dried whey to alleviate the weaning stress [3–5]. However, the milk products are so costly that some researchers compared the relative effects of ‘complex’ nursery diets containing high percentages of the milk products vs. ‘simple’ ones containing no or low percentages of the milk products on growth performance of pigs to examine the feasibility of reducing the ratio of the milk products in the nursery diets [6–10]. The rate of weight gain of nursery pigs was greater on the complex than on simple diets in all of these studies. Moreover, the difference in body weight between the two dietary groups of pigs after the nursery phase sustained or narrowed due to varying compensatory growth during the subsequent grow-finish phase. The carcass characteristics of the pigs including the backfat thickness and loin eye area at the market weight were not influenced by the nursery feeding program [8, 10]. However, the relative effects of the alternative nursery feeding programs on growth performance as well as the pig production cost under the experimental conditions were rather inconclusive. Further, little is known as to how the complexity of commercial nursery pig diets, which could be mimicked by manipulating the feeding duration on each of the nursery phase diets, influences the efficiency of pig production under commercial conditions. The present study was therefore conducted to investigate the effects of varying durations on the nursery diets on growth performance of pigs during the nursery and subsequent grow-finish phases under commercial production conditions and thereby to find the feasibility of reducing the use of the nursery diets containing the costly milk products.

## Methods

### Nursery pigs

The experimental protocol of the present study conformed to the guidelines of the Institutional Animal Care and Use Committee (IACUC) at Gyeongnam National University of Science and Technology. The animals used in the present study were handled humanely and did not receive any prolonged constraint throughout the experiment. A total of 204 (Yorkshire × Landrace) × Duroc piglets consisting of equal numbers of females and castrated males, which had been provided with 200 g of the nursery phase 2 diet/litter (Table 1) as creep feed during the last 10 d of lactation, were randomly allocated to 6 pens at weaning at 21 d of age, with three pens per sex and 34 animals per pen. One pen of females and another pen of castrated males were placed on one of three phase-feeding programs: HIGH, MEDIUM, and LOW which had varying durations on the nursery phases 1, 2, and 3 diets containing 20%, 7%, and 0% lactose and 35%, 8%, and 0% dried whey, respectively, and a grow-finish phase 1 diet (Tables 1 and 2). The lysine concentrations of the nursery diets were slightly greater than those formerly recommended by NRC [11], but were slightly less than the current NRC [12] recommendations.

**Table 1:** **Declared minimum nutritional values of the commercial nursery and grow-finish diets used in the present study (as-fed basis)**

Item	Nursery			Grow-finish			
	Phase 1 ^1^	Phase 2 ^2^	Phase 3 ^3^	High	Medium plane ^5^		
				Phase 1 ^4^	Phase 1	Phase 2	Phase 3
CP^6^, %	21.0	21.0	20.5	18.2	17.5	15.5	13.4
Lysine, %	1.40	1.40	1.25	1.10	1.0	0.80	0.70
CF^6^, %	7.5	7.5	7.0	6.0	4.0	3.0	2.5
DE^6^, Mcal/kg	3.75	3.60	3.60	3.45	3.35	3.30	3.25

^1-3^Contained 20%, 7%, and 0% lactose and 35%, 8%, and 0% dried whey, respectively.

^1-4^Used for nursery pigs between d 21 and 54 of age.

^5^Provided to grow-finish pigs from d 54 through d 182 of age.

^6^CP, crude protein; CF, crude fat; DE, digestible energy.

**Table 2:** **Nursery phase (P)-feeding programs (treatments): experimental design**

	7 days		7 days		7 days		12 days
High^1^	Nursery P 1	T^2^	Nursery P 2			T^3^	Nursery P 3
Medium^1^	Nursery P 2			T^3^	Nursery P 3		
Low^1^	Nursery P 2	T^2^	Nursery P 3			T^3^	High-plane grow-finish P 1

^1^Indicated group of nursery pigs received the indicated diets between d 21 and 54 of age. All pigs received the medium-plane grow-finish phases 1, 2, and 3 diets during days 54-96, 96-135, and 135-182 of age, respectively. See Table 1 for nutritional values of the diets.

^2,3^Transition with 20 and 30 kg of mixtures of two diets of the adjoining phases, respectively.

The HIGH group of animals received the nursery phase 1 diet to d 6 of the experiment, at which time remaining diet was removed from the feeder and three 6.7 kg portions of 2:1, 1:1 and 2:1 mixtures of the phases 1 and 2 diets, respectively, were layered sequentially on the feeder. Thereafter, the animals were provided with the nursery phase 2 diet to d 20 when a total of 30 kg of mixtures of the phases 2 and 3 diets were provided after the weigh-back as had been done at the end of d 6, followed by provision of the phase 3 diet to d 33. As such, the HIGH group animals, which received the transitive mixed diet twice approximately for 2 d each beginning from the ends of d 6 and 20, respectively, were actually on the nursery phases 1, 2, and 3 diets approximately for 7, 14, and 12 d, respectively. The MEDIUM group was provided with the nursery phases 2 and 3 diets for 14 and 19 d, respectively, whereas the LOW group was fed the nursery phases 2 and 3 and grow-finish phase 1 diets for 7, 14, and 12 d, respectively. The transitive mixed diets in the MEDIUM and LOW groups were provided at the ends of d 13 and d 6 and 20, respectively, in the same way as was used in the HIGH group. All animals were weighed by the unit of pen at the beginning and end of the nursery phase. The feed intake in each pen was measured by the weigh-back method at the ends of d 6, 13, 20 and 33.

### Growing-finishing pigs

Upon finishing the nursery feeding trial, 84 randomly selected pigs then aged 54 d (14 animals/pen) were numbered using the ear tag, weighed individually, and moved to grower pens with non-tagged pigs altogether in a pen-to-pen manner. These animals were fed the grow-finish phase 1 diet through d 96 and moved again to finisher pens where they received the grow-finish phases 2 and 3 diets successively to d 135 and 182, respectively, as described previously [13, 14]. The lysine concentrations of the grower-finisher diets, as in the nursery diets, were in between the previous and current NRC [11, 12] recommendations for corresponding stages of pigs. The grower-finisher diets were provided through a mechanical feeder line and hence the intakes of the diets were not measured. The 84 tagged animals were weighed repeatedly on d 96, 135, and 182.

A total of 60 animals (10 animals/pen), estimated carcass weights of which were within or close to the range of those eligible for the grades 1^+^ or 1 by the MAFRA [15] standards, were selected upon termination of the feeding trial, transported to a local abattoir on the following morning, given approximately 3 h of lairage, and slaughtered. The carcass weight, backfat thickness, and other carcass characteristics including the structural integrity and color of the muscle and fat, which are used as criteria for carcass grading [15], were measured or evaluated by the grading official.

### Statistical analysis

All data were analyzed using SAS (SAS Inst. Inc., Cary, NC, USA). Data obtained from nursery pigs were analyzed as a completely randomized design (CRD) using the MIXED procedure. The statistical model included the nursery feeding program and sex as a fixed effect and a random effect, respectively, with the pen regarded as the experimental unit. Data from growing-finishing animals were analyzed as a CRD with a 2 (sex) × 3 (nursery feeding program) arrangement of treatments using the GLM procedure, with the individual pig taken as the experimental unit. The initial body weights at the beginning of the nursery and grow-finish phases, which are known to be correlated with subsequent growth rates in pigs [13, 16, 17], were included in the statistical models for the growth performance variables of the nursery and growing-finishing pigs, respectively. Means were separated using the PDIFF option when a main effect or an interaction of the main effects was significant (p <0.05).

## Results

### Nursery growth performance

The initial and final body weights (BW) as well as the average daily gain (ADG) of the nursery pigs during the 33-day (d) nursery phase did not differ across the dietary treatments (Table 3). The average daily feed intake (ADFI) also did not differ among the three dietary groups during d 1-6, 1-13, or 13-33. However, the ADFI during the entire nursery phase was greater (p <0.01) in the HIGH group than in the MEDIUM and LOW groups by 20% and 17%, respectively, whereas the gain:feed ratio was not influenced by the dietary treatment.

**Table 3:** **Effects of varying phase-feeding programs on growth performance of nursery pigs**

Item	High ^1^	Medium ^2^	Low ^3^	SEM ^4^	***P***-value
Initial BW^4^, kg	5.98	5.60	5.93	0.13	0.27
Final BW^4,5^, kg	14.83 ± 0.52	13.25 ± 0.68	13.65 ± 0.46		0.42
ADG^4,5^, g	273 ± 16	225 ± 20	237 ± 14		0.42
ADFI^4^, g					
D^4^ 1-6	266	233	184	15	0.12
D 1-13	307	214	257	13	0.07
D 13-33	503	447	433	15	0.14
Overall	426^a^	355^b^	364^b^	4	0.01
Gain: feed	0.62	0.67	0.64	0.04	0.64

^1^Provided with the nursery phases 1, 2 and 3 diets for 7, 14 and 12 days, respectively.

^2^Provided with the nursery phases 2 and 3 diets for 14 and 19 days, respectively.

^3^Provided with the nursery phases 2 and 3 diets and the high-plane grow-finish phase 1 diet for 7, 14 and 12 days, respectively.

^1-3^See Table 1 for nutritional values of the diets used in each feeding program. Data are means or least squares means ± SEM of two pens consisting of 34 females and 34 castrated males, respectively.

^4^SEM, standard error of mean; BW, body weight; ADG, average daily gain; ADFI, average daily feed intake; D, day.

^5^Initial BW was included in the model as a covariate.

^a,b^Means with no common superscripts differ (p <0.05).

### Post-nursery growth

The initial BW on d 54 of age of the animals used for the continuing grow-finish feeding trial was greater (p <0.01) in the HIGH group than in the MEDIUM and LOW groups (15.5, 13.3, and 13.4 kg, respectively) as well as in barrows than in gilts (Table 4). The BW of the animals was not different among the treatments or between the two sexes at the end of the early grow-finish stage (d 96), but thereafter it was greater in the HIGH and MEDIUM groups than in the LOW group (70.3, 67.5, and 63.1 kg on d 135 and 110.0, 107.6, and 99.6 kg on d 182 for the HIGH, MEDIUM, and LOW groups, respectively; p <0.01) and also in barrows vs. gilts (p <0.05). Apart from the statistical significance, the numerical difference in BW between the HIGH and MEDIUM groups at the beginning of the grow-finish phase thus persisted through the end of the phase. In contrast, the difference between these two groups and the LOW group widened between d 96 and 182, which was largely attributable to remarkably lower BW of gilts in the LOW group compared with those in the other groups. The ADG during d 54-96, as in d-96 BW, did not differ among the treatments or between the sexes. However, the ADG during d 96-135 and 135-182 as well as overall ADG during d 54-182 were greater in the HIGH and MEDIUM groups vs. the LOW group (752, 733, and 670 g in overall ADG for the HIGH, MEDIUM, and LOW groups, respectively), but there was no difference between the former two groups. Moreover, the ADG during d 96-135 as well as overall ADG was greater in barrows than in gilts. Finally, the dressing percentage, backfat thickness, and quantitated carcass grade of the pigs slaughtered after termination of the feeding trial were not different among the treatments (Table 5).

**Table 4:** **Growth performance of the growing-finishing pigs which received the varying nursery phase-feeding programs**

Item	High ^1^		Medium ^2^		Low ^3^		***P***-value		
	B ^4^	G ^4^	B	G	B	G	FP ^4^	S ^4^	FP × S
BW^4,5^, kg									
D^4^ 54	16.3 ± 0.5	14.7 ± 0.5	14.4 ± 0.5	12.1 ± 0.5	13.1 ± 0.5	13.6 ± 0.5	<0.01	<0.01	0.03
D 96	38.8 ± 1.0	36.7 ± 0.8	35.1 ± 0.9	36.9 ± 0.9	37.3 ± 0.8	34.0 ± 0.8	0.07	0.10	0.01
D 135	73.1 ± 1.6	67.5 ± 1.5	65.7 ± 1.5	69.3 ± 1.5	67.9 ± 1.3	58.3 ± 1.4	<0.01	<0.01	<0.01
D 182	111.7 ± 3.1	108.4 ± 2.6	108.4 ± 2.7	106.8 ± 2.7	105.4 ± 2.5	93.7 ± 2.5	<0.01	0.01	0.13
ADG^4,5^, g									
D 54-96	592 ± 24	542 ± 20	503 ± 22	546 ± 22	557 ± 20	477 ± 19	0.07	0.10	0.01
D 96-135	878 ± 29	787 ± 25	787 ± 25	835 ± 26	785 ± 23	634 ± 23	<0.01	<0.01	<0.01
D 135-182	843 ± 43	876 ± 37	909 ± 37	808 ± 37	802 ± 34	756 ± 34	0.04	0.21	0.21
Overall	765 ± 24	739 ± 20	739 ± 21	727 ± 21	716 ± 20	624 ± 20	<0.01	0.01	0.13

^1^Provided with the nursery phases 1, 2 and 3 diets for 7, 14 and 12 days, respectively.

^2^Provided with the nursery phases 2 and 3 diets for 14 and 19 days, respectively.

^3^Provided with the nursery phases 2 and 3 diets and the high-plane grow-finish phase 1 diet for 7, 14 and 12 days, respectively.

^1-3^See Table 1 for nutritional values of the diets. Data are least squares means ± standard errors of means of 14 animals in each sex.

^4^B, barrow; G, gilt; FP, feeding program during the nursery phase; S, sex; BW, body weight; D, day; ADG, average daily gain.

^5^BW on d 54 was included in the model as a covariate in the analysis of subsequent BW and ADG.

**Table 5:** **Carcass characteristics of the finishing pigs which received the varying nursery phase-feeding programs**

Item	High ^1^		Medium ^2^		Low ^3^		SEM ^4^	***P***-value		
	B ^4^	G ^4^	B	G	B	G		FP ^4^	S ^4^	FP × S
Live wt^4^, kg	111.5	108.4	109.2	107.8	107.1	94.8	2.8	<0.01	0.02	0.12
Carcass wt, kg	83.3	81.4	82.4	79.1	79.9	71.5	2.1	<0.01	0.01	0.29
Dressing, %	74.8	75.1	75.7	73.4	74.7	75.4	1.0	0.89	0.63	0.27
BFT^4,5^, mm	21.4	20.9	23.8	21.9	23.2	21.9	1.3	0.38	0.22	0.86
Carcass grade^6^	2.14 ± 0.25	2.80 ± 0.30	2.00 ± 0.27	1.75 ± 0.33	2.40 ± 0.30	2.00 ± 0.66		0.14	0.99	0.23

^1^Provided with the nursery phases 1, 2 and 3 diets for 7, 14 and 12 days, respectively.

^2^Provided with the nursery phases 2 and 3 diets for 14 and 19 days, respectively.

^3^Provided with the nursery phases 2 and 3 diets and the high-plane grow-finish phase 1 diet for 7, 14 and 12 days, respectively.

^1-3^See Table 1 for nutritional values of the diets used in each feeding program. Data are means of 10 animals in each sex, except for the carcass grade in which data are least squares means ± standard errors of means.

^4^B, barrow; G, gilt; SEM, standard error of mean; FP, feeding program during the nursery phase, S, sex; wt, weight; BFT, backfat thickness.

^5^Average of the measurements between the 11 and 12^th^ ribs and at the last rib adjusted for a 110-kg live weight.

^6^Assigned 3, 2 and 1 of arbitrary point units to carcass grades 1^+^, 1 and 2, respectively. Grade 2 carcasses which did not reach the minimum weight (79 kg) eligible for the grade 1 [15] were excluded from the analysis

## Discussion

Weanling pigs, in general, are provided with a palatable milk products-based phase 1 nursery diet for a short period to maximize their intake and accordingly to minimize the post-weaning growth check resulting primarily from insufficient feed intake [1, 2, 5]. Most nursery feeding programs of commercial feed manufacturers in Korea, like the HIGH program of the present study, recommend the use of the phase 1 diet for 1 week and subsequently phases 2 and 3 diets containing lower percentages of the milk products for 2 weeks each. If the nursery pig’s intake of the milk products is restricted by providing them with diets containing no or lower percentages of milk products, their weight gain is also reduced. The reduced weight gain of nursery pigs due to nutritional insufficiency is usually made up for during subsequent grow-finish phase to varying extents depending on the animals’ genetic background and grow-finish feeding program in addition to the time, duration, and severity of the nutritional insufficiency [7, 18].

The nursery pigs which were fed the nursery phases 2 and 3 diets without (MEDIUM) or with the grow-finish phase 1 diet (LOW) ate less than those fed the nursery phase 1 diet as well as the phases 2 and 3 (HIGH). This resulted in a lesser weight gain in the former than in the latter although the nursery ADG did not differ statistically among the three groups due to the limited number of replicates (pens). The magnitude of the BW difference between the HIGH and MEDIUM groups at the beginning of the grow-finish phase persisted to the end of this phase, although final BW at termination of the feeding trial did not differ statistically between the two groups. On the other hand, according to unpublished data of Dr. B. C. Chae (Kangwon National University, Korea; personal communication), pigs weaned at 4 weeks of age gained less weight on a medium vs. high nursery feeding program similar to the present MEDIUM vs. HIGH during the nursery phase, but the pigs previously on the former caught up the growth of the others previously on the latter during the subsequent grow-finish phase. Collectively, these results suggest that whether or not the pigs after the medium vs. high nursery feeding program exhibit a compensatory growth during the grow-finish phase may also depend on the age when the animals were weaned.

The BW differential between the HIGH and LOW groups at the outset of the grow-finish phase widened with the advancing stage of the phase, which was more pronounced in gilts than in barrows. There’s no convincing explanation about this difference in post-nursery growth performance between the LOW and MEDIUM groups, because neither the ADG nor ADFI during the nursery phase was different between these two groups. It is thus speculated that the LOW program somehow impaired the growth potential of the animals probably because the planes of nutrition of the nursery phases 3 and grow-finish phase 1 diets used in this program were substantially lower than those recommended by NRC [11, 12] for nursery pigs during the stages when the diets were provided.

The MEDIUM program cost approximately 6,000 won (approximately 6 US dollar) less for nursery feed per head than the HIGH grogram. On the other hand, the MEDIUM group required approximately 4.5 kg of additional grow-finish diet for 1.5 kg of additional weight gain and also 3 more days on feed costing 2,250 and 750 won per head, respectively, than the HIGH group to reach a 110-kg market weight, assuming that the feed cost per kilogram weight gain during the entire growth-finish phase was equal in both groups. The total production cost per 110-kg market pig was thus assessed to be saved approximately 3,000 won by substitution of the MEDIUM nursery feeding program for the commonly used HIGH program. Relative production cost per market pig for the LOW group, however, could not be extrapolated, because the growth performance of this group during the grow-finish period was much lower than that of the other groups.

## Conclusions

Results of the present study indicate that substitution of the MEDIUM nursery feeding program using only the nursery phases 2 and 3 diets for the common HIGH program using the phase 1 diet in addition to the phases 2 and 3 causes a reduced weight gain of nursery pigs and that the reduced BW due to the substitution persists throughout the grow-finish phase. Nevertheless, the former seems to be more efficient than the latter in terms of production cost per market pig whereas the LOW program using the nursery phases 2 and 3 and grow-finish phase 1 diets is thought not to be worthy of further consideration because of its severely negative effect on post-nursery pig growth. Obviously, however, more studies are necessary to confirm the present assessment.

## References

[1] Weary DM, Jasper JJ, Hotzel MJ (2008). Understanding weaning stress. Appl Anim Behav Sci.

[2] Kim JC, Hansen CF, Mullan BP, Pluske JR (2012). Nutrition and pathology of weaner pigs: nutritional strategies to support barrier function in the gastrointestinal tract. Anim Feed Sci Technol.

[3] Maxwell CV, Carter SD, Lewis AJ, Southern LL (2001). Feeding the weaned pig. Swine Nutrition.

[4] DeRouchey JM, Goodband RD, Tokach MD, Nelssen JL, Dritz SS (2010). Nursery swine nutrient recommendations and feeding management. National Swine Nutrition Guide.

[5] Kim SW, Hansen JA, Chiba LI (2013). Diet formulation and feeding programs. Sustainable Swine Nutrition.

[6] Tokach MD, Pettigrew JE, Johnston LJ, Overland M, Rust JW, Cornelius SG (1995). Effect of adding fat(or) milk products to the weanling pig diet on performance in the nursery and subsequent growth-finish stages. J Anim Sci.

[7] Whang KY, McKeith FK, Kim SW, Easter RA (2000). Effect of starter feeding program on growth performance and gains of body components from weaning to market weight in swine. J Anim Sci.

[8] Wolter BF, Ellis M, Corrigan BP, DeDecker JM, Curtis SE, Parr EN, Webel DM (2003). Impact of postweaning growth rate as affected by diet complexity and space allocation on subsequent growth performance of pigs in a wean-to-finish production system. J Anim Sci.

[9] Chae BJ (2013). Strategies for saving the feed cost by improving the feed efficiency in pig production (in Korean). Proceedings of "Symposium on Strategies for Reducing Production Cost of Animal Production", organized by Korean Society of Animal Nutrition & Feedstuffs during 2013 Annual Congress of Korean Society of Animal Sciences and Technology.

[10] Skinner LD, Levesque CL, Wey D, Rudar M, Zhu J, Hooda S, de Lange CFM (2014). Impact of nursery feeding program on subsequent growth performance, carcass quality, meat quality, and physical and chemical body composition of growing-finishing pigs. J Anim Sci.

[11] NRC (1998). Nutrient Requirements of Swine.

[12] NRC (2012). Nutrient Requirements of Swine.

[13] Ha D-M, Jang K-S, Won H-S, Ha S-H, Park M-J, Kim SW, Lee CY (2011). Effects of creep feed and milk replacer and nursery phase-feeding programs on pre- and post-weaning growth of pigs. J Anim Sci Technol.

[14] Ha D-M, Jung D-Y, Park MJ, Park B-C, Lee CY: **Effects of sires with different weight gain potentials and varying planes of nutrition on growth of growing-finishing pigs.** *J Anim Sci Technol* In press 10.1186/2055-0391-56-22 PMC4540266 26290711

[15] MAFRA (2014). Grading Standards for Livestock Products (in Korean). Notification No. 2014–4 of the Ministry of Agriculture, Food and Rural Affairs, Republic of Korea.

[16] McConnell JC, Eargle JC, Woldorf RC (1987). Effects of weaning weight, co-mingling, group size and room temperature on pig performance. J Anim Sci.

[17] Mahan DC, Lepine AJ (1991). Effect of pig weaning weight and associated nursery feeding programs on subsequent performance to 105 kilograms body weight. J Anim Sci.

[18] Lawrence TLJ, Fowler VR, Novakofski JE (2012). Growth of Farm Animals.

